# Tough magnesium phosphate-based 3D-printed implants induce bone regeneration in an equine defect model

**DOI:** 10.1016/j.biomaterials.2020.120302

**Published:** 2020-08-23

**Authors:** Nasim Golafshan, Elke Vorndran, Stefan Zaharievski, Harold Brommer, Firoz Babu Kadumudi, Alireza Dolatshahi-Pirouz, Uwe Gbureck, René Van Weeren, Miguel Castilho, Jos Maldaa

**Affiliations:** a Department of Orthopedics, University Medical Center Utrecht, GA, Utrecht, the Netherlands; b Regenerative Medicine Utrecht, Utrecht, the Netherlands; c Department for Functional Materials in Medicine and Dentistry, University of Wurzburg, Germany; d Department of Equine Sciences, Faculty of Veterinary Sciences, Utrecht University, the Netherlands; e Technical University of Denmark, Department of Health Technology, 2800 Kgs, Lyngby, Denmark; f Technical University of Denmark, Department of Health Technology, Center for Intestinal Absorption and Transport of Biopharmaceuticals, 2800 Kgs, Lyngby, Denmark; g Department of Regenerative Biomaterials, Radboud University Medical Center, Philips van Leydenlaan 25, Nijmegen, 6525 EX, the Netherlands; h Orthopedic Biomechanics, Department of Biomedical Engineering, Eindhoven University of Technology, Eindhoven, the Netherlands

**Keywords:** 3D printing, Bone tissue engineering, Composites, Magnesium phosphates, Strontium, *In vivo*, Osteoinduction

## Abstract

One of the important challenges in bone tissue engineering is the development of biodegradable bone substitutes with appropriate mechanical and biological properties for the treatment of larger defects and those with complex shapes. Recently, magnesium phosphate (MgP) doped with biologically active ions like strontium (Sr^2+^) have shown to significantly enhance bone formation when compared with the standard calcium phosphate-based ceramics. However, such materials can hardly be shaped into large and complex geometries and more importantly lack the adequate mechanical properties for the treatment of load-bearing bone defects. In this study, we have fabricated bone implants through extrusion assisted three-dimensional (3D) printing of MgP ceramics modified with Sr^2+^ ions (MgPSr) and a medical-grade polycaprolactone (PCL) polymer phase. MgPSr with 30 wt % PCL (MgPSr-PCL30) allowed the printability of relevant size structures (>780 mm^3^) at room temperature with an interconnected macroporosity of approximately 40%. The printing resulted in implants with a compressive strength of 4.3 MPa, which were able to support up to 50 cycles of loading without plastic deformation. Notably, MgPSr-PCL30 scaffolds were able to promote *in vitro* bone formation in medium without the supplementation with osteo-inducing components. In addition, long-term *in vivo* performance of the 3D printed scaffolds was investigated in an equine tuber coxae model over 6 months. The micro-CT and histological analysis showed that implantation of MgPSr-PCL30 induced bone regeneration, while no bone formation was observed in the empty defects. Overall, the novel polymer-modified MgP ceramic material and extrusion-based 3D printing process presented here greatly improved the shape ability and load-bearing properties of MgP-based ceramics with simultaneous induction of new bone formation.

## 1 Introduction

Given the diversity of the treated clinical pictures (ranging from infantile craniofacial anomalies to trauma or cancer), medical progress, and population ageing, a 10% annual increase of bone grafting procedures is expected [1]. To satisfy the growing need for bone repair, the development of new biomaterials and fabrication methods has received great attention. Degradable scaffolds can be either ceramic (e.g.hydroxyapatite [2,3], tri-calcium phosphate [4], or bioglass [5]), polymer-based (e.g. polycaprolactone [6], polylactide-co-glycolide [7]) or composites of both classes of material [8]. These materials have been produced by different conventional fabrication methods ranging from porogen leaching [9], freeze-casting [10,11] to casting and gas foaming [12]. Due to their poor shape ability and limited mechanical properties of these materials, one of the biggest challenges remains the generation of scaffolds for the treatment of larger and complex defects (typically above 10 mm) [13]. One promising approach to address the above-mentioned limitations is through extrusion assisted three-dimensional (3D) printing of ceramics. 3D printing allows the generation of engineered bone scaffolds from a computer-aided design (CAD) model. Numerous 3D printing techniques have been developed to fabricate tailored bone scaffolds. The most investigated techniques for 3D printing of bioceramics involve 3D powder printing [14–17], low temperature [18,19] and high temperature [8,20] extrusion-based 3D printing. Previously, Adam et al. manufactured hyper-elastic scaffolds for bone repair composed of hydroxyapatite and polycaprolactone or poly (lactic-co-glycolic acid), using extrusion based 3D printing at room temperature, which is a versatile technology for pasty materials [8]. However, printed purely ceramic materials generally lack load-bearing properties. However, the polymer-ceramic composites may have improved mechanical properties, but feature-limited osteoinductivity due to polymer masking and lower solubility of the ceramic phases, such as hydroxyapatite (HA), tri-calcium phosphate (TCP), calcium-deficient hydroxyapatite (CDHA), and biphasic calcium phosphates (BCPs). Recently, magnesium phosphate cement (MPC) or metal ions into calcium phosphate cement (CPC) were introduced, showing a great promise for stimulating bone formation [21,22]. Recently, magnesium phosphate (MgP) materials have captured increasing attention due to their high *in vivo* solubility and low tendency to transform into lower soluble CaP phases at physiological conditions [23]. In addition, the incorporation of osteopromotive ions, like Sr^2+^, into CaP and MgP materials has been shown to induce new bone formation [14,24–26].

Here, we have developed individually shaped 3D printed magnesium phosphate scaffolds with controlled mechanical and biological properties. Control over mechanical and biological properties was obtained by incorporation of an elastic medical-grade PCL phase, a widely accepted thermoplastic material for bone repair [27], and low dosages of biologically active Sr^2+^ ions, respectively. The effect of PCL and Sr^2+^ on MgP printability was evaluated by filament collapse and fusion testing. In addition, the structural and mechanical properties of the printed composite were investigated. The biological effect of PCL and Sr^2+^ on MgP ceramic bone-forming potential was evaluated *in vitro* over 28 days in both basal and osteogenic medium firstly and then followed by a long-term (6 months) *in vivo* experiment in an equine tuber coxae defect model.

## 2 Materials and methods

### 2.1 MgP-based ceramics powders preparation

MgP based powders for 3D printing were synthesized as previously described [28]. Briefly, a homogenous mixture of reactants composed of magnesium hydrogen phosphate (MgHPO_4_.3H_2_O, Sigma-Aldrich, Steinheim, Germany), magnesium hydroxide (Mg(OH)_2_, VWR International GmbH, Darmstadt, Germany), and strontium carbonate (SrCO_3_, Sigma-Aldrich, Steinheim, Germany) in appropriate molar ratios (Table 1) were homogeneously mixed in a planetary ball mill (PM400, Retsch GmbH, Haan, Germany) for 1h at 200 U/min using 4 agate balls (d_ball_ = 30 mm). Thereafter, these powder mixtures were sintered at 1050 °C for 5 h. Afterwards, the sintered cakes were crushed with a pestle in a mortar followed by wet grinding in 100% ethanol for 2 h in the planetary ball mill (180 ml ethanol, 125 g cement, 250 U/min) using 200 agate balls (d_ball_ = 10 mm). In a final step, the cement powders were decanted and dried at room temperature.

### 2.2 Paste preparation and extrusion-based 3D printing

Extrusion pastes were prepared by combining prepared Mg_2.33_Sr_0.67_(PO_4_)_2_ powder (MgPSr) and commercial medical grade Poly (ε-caprolactone) (mPCL, Purasorb PC 12, Purac Biomaterials, Netherlands) in different weight ratios of MgPSr to PCL (70:30, 60:40, and 50:50 wt%). The initial materials were dissolved in a mixture of high volatile solvents composed of dichloromethane (Sigma-Aldrich, Germany), 2-BU-1-(4- (diethylamino) anilino)-3-me-pyrido(1,2-a) benzimidazole - 4 - carbonitrile (Sigma-Aldrich, Germany), and dibutyl phthalate (Sigma-Aldrich, Germany) in a ratio of 10:2:1 wt%. After the mixture of 90 wt% powder with the solvent as described above, pastes were left homogenizing for 24h on a roller mixer at 37 °C.

According to the amount of polymer (100, 50, 40, and 30 wt%), different compositions were identified as PCL alone, MgPSr-PCL50, MgPSr-PCL40, and MgPSr-PCL30, respectively. This nomenclature will be used throughout the manuscript.

**Fig. 1:**
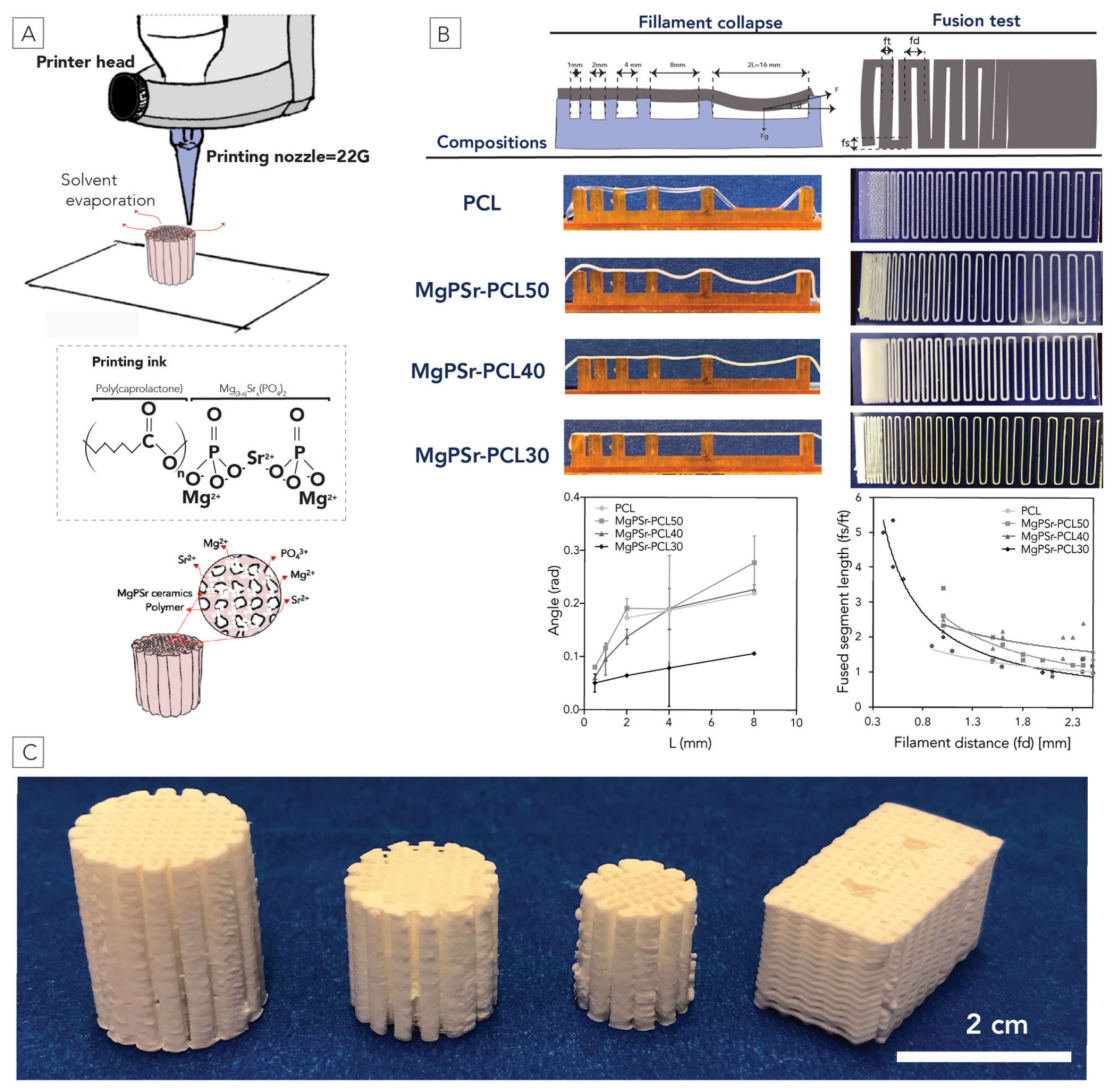
Preparation and printability characterization of the bioactive materials. A) Schematic illustration of the low-temperature printing process and the composition of the ink. B) Printability evaluation. Filament test: different compositions extruded over pillar support with different spacings. Effect of ceramics concentration on the angle of deflection θ, in radians, as a function of half the gap distance L, in mm (n = 3 for each group). Fusion test: Pictures from stereo microscopy after deposition on the glass slides (n = 3). The exponential fitting of the fused filament length is normalized by filament thickness as a function of the filament distance for the tested compositions. C) Designed and printed scaffolds with various shapes of MgPSr-PCL30.

MgP based scaffolds were fabricated using an extrusion-based 3D printing system (3D Discovery, regenHu, Switzerland). Initially, pastes were transferred to a 5 mL syringe (Nordson EFD, USA) and extruded through a 22G conical nozzle, 0.41 mm (Nordson EFD, USA) (Fig. 1A). Continuous paste deposition was achieved by applying a dispensing pressure of 0.9 bar. Printability of the different pastes was first evaluated by a filament collapse and fusion test according to a protocol described elsewhere [29]. Briefly, for the filament collapse test, a single paste filament was deposited onto a platform with pillars spaced by 1.0, 2.0, 4.0, 8.0, and 16.0 mm using extrusion parameters as mentioned before. Filament sagging was quantified by measuring the angle of deflection (θ) at the edge of the suspended filament using image J software (version 1.51k, NIH, USA), as illustrated in Fig. 1B at various gaps. For the filament fusion test, pastes were printed in meandering patterns composed of parallel strands at increasing spacings, from 0.1 mm to 2.5 mm, and increasing 0.2 mm for each subsequent line (Fig. 1B). After taking pictures, the fused segment length (fs) at each filament distance (fd) were measured using ImageJ and normalized by dividing fs by the average of filament thickness (ft) to avoid the effect of filament thickness variation between different concentrations of PCL. All measurements were repeated 3 times and the images were recorded by a stereomicroscope (Olympus SZ61, magnification 4.2x, resolution 2040 x1536 pixels) immediately after printing. Afterwards, cylindrical shape scaffolds with different sizes (d = 10 mm and h = 10, 15 mm), and rectangular (10 mm, 10 mm, and 20 mm) with a defined pore size of 1 mm were fabricated to further analysis.

### 2.3 Physical and chemical characterization

The morphology of the synthesized ceramic powders and the microstructures of the 3D-printed constructs were analyzed using a Scanning Electron Microscope (XL30SFEG, FEI, USA) at an acceleration voltage of 10 kV. Prior to imaging, all samples were coated with gold with a thickness of 6 nm. In addition, the phase composition of both synthesized powders was determined by X-ray diffraction (Bruker AXS, Germany) using monochromatic Cu-Kα radiation. Measurements were collected from 2*θ* = 20–40° with a step size of 0.02°. The phase composition of homemade powders was checked by JCPDS reference patterns (Mg_3_(PO)_4_, Farringtonite, PDF ref. 00-033-0876) and Magnesium Strontium Phosphate (Mg_2_Sr(PO_4_)_2_, PDF ref. 00-014-0206).

The porosity of the printed scaffolds was characterized by micro-CT analysis using a Quantum FX-Perkin Elmer (μCT, Quantum FX, PerkinElmer, USA). Constructs were scanned at 90 kV tube voltage, 180 mA tube current, 30 μm resolution and 3 min scan time. Volume calculation and the porosity of the printed scaffolds were determined by measuring trabecular parameters in 3D μCT images according to the suggested protocol [30]. Briefly, slices scan of the scaffolds was opened and the local threshold was adjusted based on Bernsen and Niblack’s thresholding method using ImageJ software. Finally, bone volume fraction (BV/TV) and porosity (1- (BV/TV)) were measured with BoneJ plugin for the circular region of interest (ROI).

### 2.4 Mechanical characterization of printed constructs

Uniaxial compression tests were performed using a universal testing machine (Zwick Z010, Germany) equipped with a 1 kN load cell. Quasistatic tests were performed at a rate of 1 mm/min, in the air at room temperature, according to a protocol described elsewhere [16]. Tests were conducted on cylindrical samples (d = 6 mm, h = 12 mm, n = 5) with a pore size of 1 mm. From the quasi-static measurements, the elastic modulus (defined as the slope of the linear region from 0.02 to 0.05 mm/mm), the yield stress (defined as the point where nonlinear deformation begins), and toughness (defined as the absorbed energy by the scaffolds up to yield stress) were determined. In addition, to access the elastic behavior of the printed constructs, dynamic compression tests were conducted on MgPSr-PCL30 samples (n = 3). Tests were performed by applying a ramp force to a height of 2 mm (equivalent to a strain of 2%) followed by a sinus wave deformation at a frequency of 0.1 Hz. From the dynamic tests, the compression loading profiles of MgPSr-PCL30 scaffolds in the strain and time-domain were determined. Uniaxial tensile testing was performed on rectangular-shaped MgPSr-PCL30 based scaffolds (l = 60 mm, w = 10 mm, and t = 1 mm). Tests were conducted at a rate of l mm/min at room temperature. From the engineered stress-strain curves, elastic modulus, yield stress, and toughness were determined. Tensile elastic modulus was determined as the slope of the linear part of the curve between 0.02 and 0.03 mm/mm, while yield stress and toughness were determined as stated above for the uniaxial compression tests.

### 2.5 Lipase accelerated degradation experiments

MgPSr-PCL30 printed implants were incubated in a 0.4 mg/ml lipase solution (from *Pseudomonas cepacian*, Sigma-Aldrich, Germany) and 1 mg/ml sodium azide (Sigma-|Aldrich, Germany). Incubation was performed at 37 °C for 15 days (with intermediate time points 1, 5, 10 and 15 days) and media were refreshed every 4 days. As a control, scaffolds were also incubated in PBS alone. At each time point, samples were monitored for weight loss (quantified as $\frac{W_{D15}-W_{0}}{W_{0}}\times 100$) and compressive mechanical properties. Compression tests were performed according to section 2.4. Before mechanical testing, scaffolds were washed thoroughly with Mili-Q water and dried in a desiccator for 2 days.

### 2.6 Ion release study

The release profile of magnesium, phosphorous and strontium ions from the 3D-printed scaffolds was recorded utilizing Inductively Coupled Plasma Mass Spectrometry (ICP-MS, Varian, Darmstadt, Germany) during 21 days. Samples (disc shape with a diameter of 10 mm and thickness of 3 mm) were immersed in 5 ml Mili-Q water and 0.1M Tris-HCl (Tris(hydroxymethyl)aminomethane, Sigma-Aldrich, Germany) at 37 °C. To quantify the concentration of released ions, the solutions were 10X diluted in 1.3 v/v% HNO_3_ (65% Suprapur, Merck, Schwalbach, Germany) and measured against standard solutions (Merck, Schwalbach, Germany, Ca^2+^: 0.5 ppm and 1 ppm, Mg^2+^: 1 ppm, 5 ppm, P^-^: 100 ppm and 500 ppm, Sr^2+^: 10 ppm and 200 ppm). The ion concentrations at each timepoint were calculated relative to the amount of fresh medium and the cumulative concentration of released ions was reported over 21 days. To compare the ion release of MgPSr-PCL30, the MgP-PCL30 scaffolds were used as control.

### 2.7 In vitro cell culture

To ensure effective removal of solvents all scaffolds used for in vitro cell experiments were washed for 6 h in 70 v/v% ethanol in water followed by 5 times washing with Mili-Q water for 24h. After washing, samples were sterilized for 2h under ultraviolent (UV) light and subsequently immersed in cell culture media supplemented with 1v/v% penicillin/streptomycin (Pen-Strep) (all Gibco, Thermo Fisher, USA) for 3 days to remove any remaining solvents from the printing process. Equine mesenchymal stem cells (eMSCs) were harvested from healthy bone marrow aspirates according to a protocol described elsewhere [31]. EMSCs were then first expanded 7 days in α-MEM supplemented with 10% (v/v) fetal bovine serum (FBS), 0.2 mM L-ascorbic-acid-2-phosphate (ASAP), and 1% (v/v) Pen-Strep at 37 °C in a humidified atmosphere containing 5% CO2, and then seeded (passage number = 3) onto scaffolds (PCL, MgPSr-PCL30, and MgP-PCL30) at a density of 30, 000 cells per cm^2^. Cell-laden constructs were cultured in basal media for 7 days, then divided into two groups: samples cultured in 1) basal medium (α-MEM+10% FBS+0.2 mM ASAP+1% Pen-Strep, Sigma-Aldrich, Germany) and in 2) osteogenic medium (α-MEM+10% FBS+0.2 mM ASAP+1% Pen-Strep+ 10 nM Dexamethasone,+10 mM B-glycerophosphate, Sigma-Aldrich, Germany). Medium was changed every 3 days, and at least 3 scaffolds were tested per group.

### 2.8 Cytocompatibility and osteogenic potential evaluation

Cell viability of MgP based scaffolds was determined using a live-dead viability kit for mammalian cells (Invitrogen Life Technologies, USA), according to the manufacturer’s instructions and a protocol described elsewhere [32]. Stained cell-laden constructs were imaged with a confocal microscope (Leica SP8X Laser Scanning, Germany) with 494 nm (green, Calcein) and 528 nm (red, EthD-1) excitation filters, and at least 3 samples were analyzed per group. Images of the whole scaffolds were merged using the mosaic function of the Leica LASX software. Moreover, the distribution of live and dead cells relative to the scaffold was quantified using Adobe Photoshop cc 2019. Cell metabolic activity was quantified by Alamar blue, following the manufacturer’s instruction, while DNA content was measured using a Quant-iT-Picogreen-dsDNA kit (Molecular Probes, Invitrogen, Carlsbad, USA).

The osteogenic differentiation of the cells was measured using alkaline phosphatase (ALP) and Alizarin Red staining (ARS) as early and late osteogenic markers, respectively. The alkaline phosphatase assay was performed after lysis of the cells in TE-buffer (Tris-EDTA buffer, 10 mM Tris-HCl, 1 mM EDTA, pH 8) after 1 and 7 days of culturing. The cell-laden constructs were thawed and frozen 3 times to lyse the cells in TE-buffer. The alkaline phosphatase activity was measured using the conversion of the p-nitrophenyl phosphate liquid substrate System (pNPP, Sigma-Aldrich). The standard ALP measurements using serial dilutions of calf intestinal ALP (Sigma-Aldrich, Germany) in TE-buffer was used to normalize the measured ALP values. The samples were incubated on the shaker for 30 min and every 5 min, the absorbance was measured at 405 nm and corrected at 655 nm (Bio-rad, Hercules, CA, USA). Results were normalized to DNA content from the same cell lysate used to measure ALP, using a Quan-iT-Picogreen-dsDNA kit (Molecular Probes, Invitrogen, Carlsbad, USA) following the manufacturer’s instructions.

Calcium deposition was determined by Alizarin red staining (ARS) (2% solution, pH 4.2, Sigma-Aldrich) staining at 21- and 30-day time points as described elsewhere [33]. Prior to incubation of cell-laden constructs with ARS solution, the samples were fixed in 4% formaldehyde. Calcium deposits were visualized by stereomicroscope (Olympus SZ61, magnification 1.5x). For quantification of calcium deposition, 35 mg/ml fresh cetylpyridinium chloride (CPC) solution in DI water (pH = 7.4–7.8) was added to the stained samples under agitation for 30 min at 37 °C. It was then read with a UV/vis plate reader at 405 nm. The absorbance of ARS concentration conversion was done using a pre-plotted standard curve. Moreover, to confirm the calcium deposition within the extracellular matrix, after 30 days of culture, the cell-laden scaffolds were washed with deionized water and fixated in 2.5% Glutaraldehyde (Sigma-Aldrich, Germany).

To analyze the chemical structure of the seeded scaffolds, Fourier-Transform Infrared spectra were obtained in the range of 4000 - 500 cm^–1^ at a resolution of 4 cm^–1^ using an FTIR spectrometer (PerkinElmer Spectrum 100, USA) fitted with a diamond crystal attenuated total reflectance (ATR). Spectrum signals were averaged over 16 scans, and the obtained spectra were baseline-corrected and normalized using the PerkinElmer Spectrum software. X-ray diffraction patterns of the seeded scaffolds after 30 days of culturing were recorded at room temperature using Cu Kα1 radiation (λ = 1.54056 Å) and Ge (111) monochromator operating at 40 kV and 40 mA (HUBER G670 Guinier imaging-plate detector powder diffractometer, Germany). XRD patterns were collected with 2θ ranging from 20° to 50° and a scan step size of 0.005°. The morphology of eMSCs on 3D printed scaffolds were observed using backscatter scanning electron microscope (voltage 10 kV). To fix the cells, at specific time points, the scaffolds immersed in 2.5 vol% Gluta-rdehyde (Sigma-Aldrich, Germany) for 1 h and then dehydrated in gradient ethanol solution (each step 10 min). Afterwards, the scaffolds were kept in a desiccator prior to imaging. Additionally, after 14 and 21 days of *in vitro* experiment, all the samples kept in formalin (4%), embedded in Agarose (4w. %, Sigma-Aldrich, Germany) and cut through the top of the scaffolds. Osteonectin a major non-collagenous protein in bone (Osteonectin AB SPARC AON-1, DSHB; nuclei: blue, positive osteonectin: brown) and collagen type 1 (Anti-collagen I antibody EPR7785, Abcam; nuclei: blue, positive collagen type I: brown) were performed to reveal the activity of osteoblasts.

### 2.9 Animal experiments

In total, 8 adult female ponies (age 5–14 years, mean body weight 173 ± 38 kg) were used for this study. Surgery was performed in a standing position, under local anaesthesia and sedation (detomidin (Domosedan®, 10 mcg/kg) + morphine (0.1 mg/kg) intravenously applied via a jugular catheter in combination with local infiltration with mepivacaine (Mepidor), 10 mL/site)). A critical-sized defect was created in both tuber coxae of the ponies. In detail, a vertical incision was created through the skin, subcutis and periosteum onto the bone of the tuber coxae. Subsequently, the periosteum was dissected from the bone. A drill hole of 11 mm diameter and 10 mm deep was created using an orthopaedic drill and drill sleeve. The cylindrical MgPSr-PCL30 scaffolds (d = 10 mm, h = 10 mm) were implanted at one side while the contralateral tuber coxae remained empty as a control. The surgical wounds, including periosteum, the overlying fat, subcutis and skin were closed in 3 layers using synthetic resorbable suture material post-operatively, clinical parameters such as degree of lameness, discomfort, and temperature were monitored daily during 4 weeks while the animals were kept in boxes. NSAIDs were given for 5 days (meloxicam, Metacam®, per os, 0.6 mg/kg bwt). Subsequently, the animals received pasture exercise for 5 months. After six months, the ponies were sacrificed, and the tuber coxae were harvested and fixed in formalin for processing and further analysis. The animal study was approved by the Instantie voor Dierenwelzijn Utrecht (IvD, Utrecht Animal Welfare Body) and complied with international recommendations for care and use of laboratory animals (approval ethical number AVD108002015307).

### 2.10 Micro-computed tomography

To visualize the calcified tissue at the defect site, the harvested tissue underwent micro-CT analysis (Quantum FX-Perkin Elmer, USA). The scan parameters were 90 kV tube voltage, 180 mA tube current, 40 mm resolution and 3 min scan time. Bone ingrowth and degradation of the scaffolds were quantified using BoneJ plugin and ImageJ based on the thresholding method specified in point 2.3.

### 2.11 Histology and immunohistochemistry

The harvested constructs were cut into two parts; one half was embedded in polymethylmethacrylate (MMA, Sigma-Aldrich, Germany) and the other half was slowly decalcified in formalin and ethylenedi-amine tetra-acetic acid (EDTA, Sigma-Aldrich, Germany) for 3 months and subsequently embedded in paraffin. Samples embedded in MMA were sectioned in 300–400 μm slices using a Leica 4 SP1600 Saw Microtome system (Leica, Germany). After sectioning, the samples were stained with methylene blue/basic fuchsine and evaluated with light microscopy (Olympus BX51, Japan) [34]. Paraffin-embedded samples were sectioned using a microtome (n = 6) (Leica sawing microtome, Nusslochh, Germany) in 5 μm slices and stained with hematoxylin and eosin (H&E staining, thermo Fisher scientific, USA) for tissue overview analysis, and picro-sirius red staining (Thermo Fisher Scientific, USA) for collagen analysis. Collagen orientation was visualized with polarized light (Olympus BX51, Japan). Backscatter images using a Secondary Electron Detector were analyzed by EDX with a scanning electron microscope (FEI XL30SFEG, USA). Before EDX analysis for newly formed bone and native bone, MMA sections were polished and sputtered with gold.

### 2.12 Statistical analysis

Data were represented as mean ± standard deviation. The significance of differences between the groups for the different printability and mechanical parameters was assessed using a one-way ANOVA and post hoc Tukey’s test (Graphpad prism V8). Differences were considered significant at a probability error (p) of p ˂ 0.05.

## 3 Results

### 3.1 Extrusion printing of magnesium phosphate-based materials

MgP-based powders substituted with Sr^2+^ ions were thermally synthesized and then milled to achieve particle sizes suitable for extrusion-based printing, *i.e*. between 2 and 5 μm, (Figs. S1A and B). X-ray diffraction confirmed the purity of MgP and successful incorporation of Sr^2+^ ions (Fig. S1C). The 3D printed scaffolds composed of MgPSr and PCL were successfully fabricated by extrusion-based printing at room temperature (Fig. 1A). The printability of the MgPSr powder modified with different amounts of PCL polymer was quantitatively assessed via filament collapse and stackability test. The filament sagging angle decreased with less polymer content, which revealed that the decrease of polymer content resulted in a more viscous paste, improving filament printability (Fig. 1B). Furthermore, the filament fusion tests show that a minimum inter-fiber spacing of 0.5–0.8 mm could be achieved for MgPSr-PCL30 (Fig. 1B). For the other compositions such as MgPSr-PCL40 and MgPSr-PCL50, an increase in fd was observed for shorter fs distances. A non-linear inverse relation between fs and fd was observed, which is also clear from the meandering printed patterns (Fig. 1B). To further explore the printing flexibility and scalability of this composite material, MgPSr-PCL30 composition was printed in cylindrical and rectangular shapes constructs of different sizes as described in part 2.2 (Fig. 1C).

### 3.2 Mechanical characterization of 3D printed magnesium-based scaffolds

**Fig. 2:**
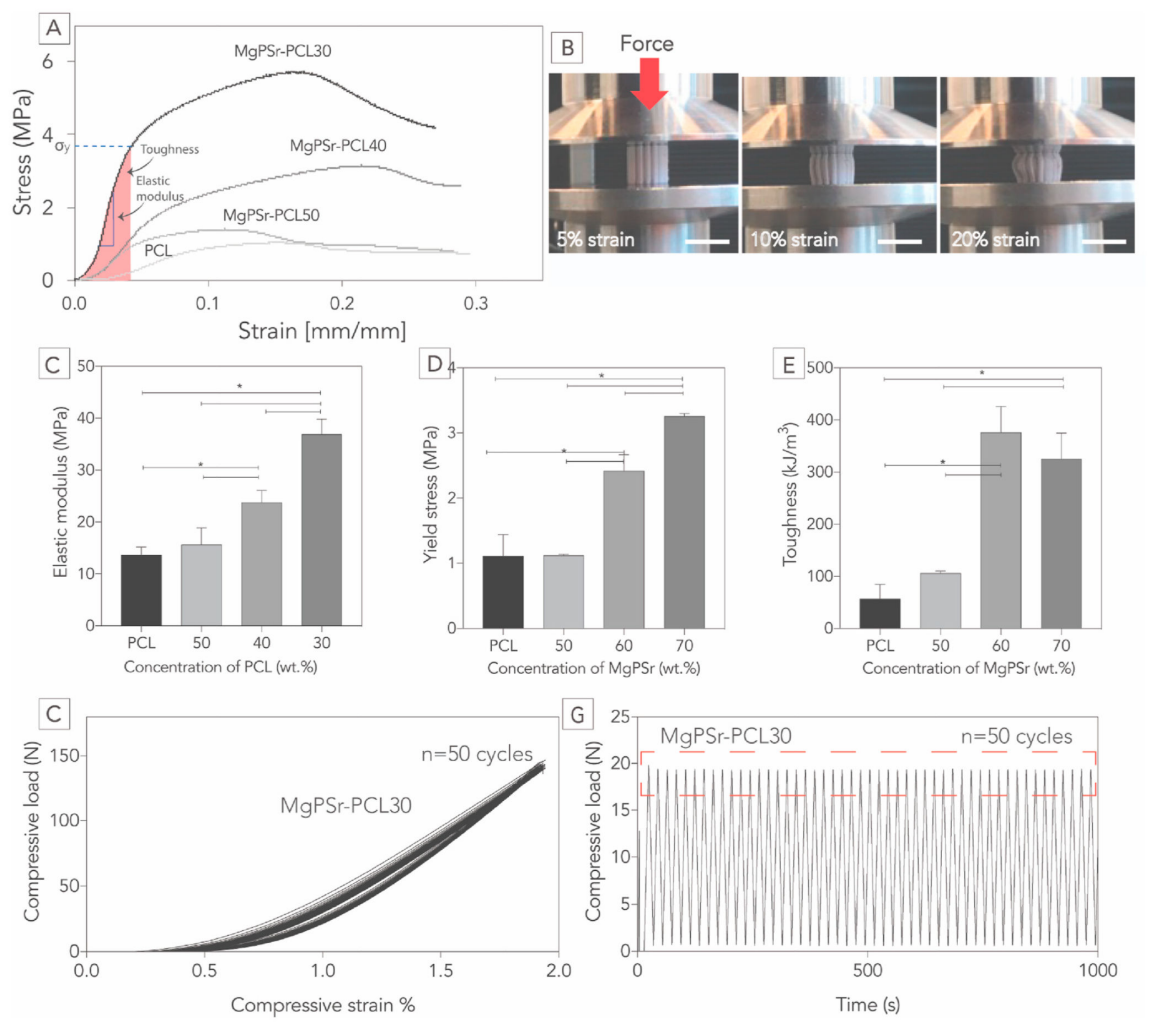
Evaluation of the static and dynamic mechanical properties. A) Longitudinal compression profile of 3D printed scaffolds with various rations of PCL. B) Corresponding photographs showed the plastic deformation of the scaffolds begins at 10% strain and proceeds to buckle and barrel. Interpreted data (scale bar 10 mm) C) Elastic modulus, D) Yield stress, and E) Toughness from compressive loading profile for various concentrations of PCL. The compression and recovery profile of 3D printed scaffolds (50 cycles) in F) strain domain and G) time domain for MgPSr-PCL30 scaffolds. Additional information on mechanical properties can be found in the Supplementary Information.

**Fig. 3:**
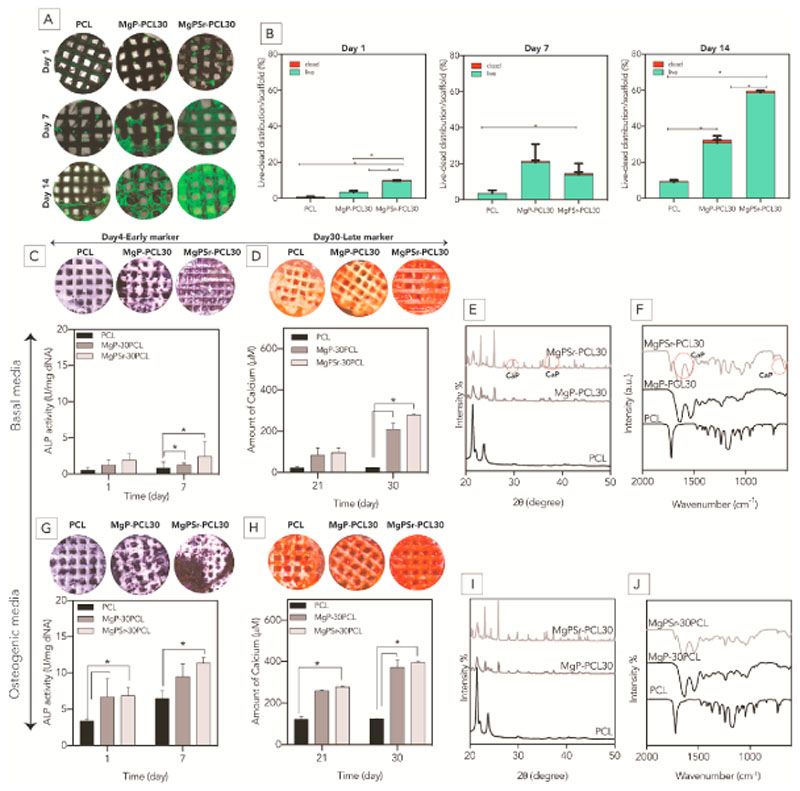
*In-vitro* assessment of bioactivity of the printed scaffolds. A) confocal images from the live-dead staining assay during 14 days culturing of eMSCs in basal media. B) Quantified results of the distribution of live and dead cells per scaffold. C and G) alkaline phosphatase (ALP) images of the printed samples. ALP activity levels were normalized to DNA content. D and H) Formation of the calcified matrix by eMSCs investigated using Alizarin Red S staining after 30 days of culture. Quantified amount of calcified matrix evaluated by Alizarin Red S content on the 3D printed scaffolds using a colorimetric assay. Scale bars represent 1 mm. E and I) XRD and F and J) FTIR analysis of the scaffolds after culturing of eMSCs. Peaks corresponding to hydroxyapatite are marked with red circles. (For interpretation of the references to color in this figure legend, the reader is referred to the Web version of this article.)

Addition of a PCL phase overcame the brittleness of MgP ceramics modified with Sr^2+^ ions. All the ceramic-polymer composite scaffolds showed similar stress-strain behavior as the pure PCL scaffolds alone (Fig. 2A and B). The increase of polymer content resulted in a decrease in both elastic modulus and yield stress of the composites. For instance, the elastic modulus and yield stress increased for MgPSr-PCL30 4.5 and 2.7 times, respectively, when compared to PCL scaffolds alone (Fig. 2C and D) reaching values of 36.8 ± 2.9 MPa and 4.3 ± 0.1 MPa, respectively. Moreover, the determining elastic modulus for MgPSr-PCL30 was 1.5 and 2.3 times higher than those observed for MgPSr-PCL40 and MgPSr-PCL50, respectively. After the addition of 40 and 30 PCL% to the MgPSr ceramic phase, composite scaffolds reached a compressive toughness of 375.5 ± 50.1 kJ/m^3^ and 324.8 ± 50.0 kJ/m^3^ respectively (Fig. 2E). Notably, MgP composite scaffolds allowed easy handling and shaping, which is crucial for orthopaedic surgeons to allow optimal accommodation of implants to the defect (Fig. S2). This elastic behavior of the composite scaffolds was further evaluated by performing uniaxial tensile tests. MgPSr-PCL30, the composition that allowed a high printing resolution combined with high compressive toughness (324.8 ± 50.0 kJ/m^3^), was also able to deform up to 10% tensile strain without failure and showed values for tensile yield stress (1.5 ± 0.4 MPa) and toughness (24.8 ± 13.2 kJ/m^3^) in the range of pure PCL scaffolds [35]. This elastic behavior was confirmed by evaluating permanent deformation under cyclic compression at 2% deformation. MgPSr-PCL30 composites could resist compressive forces of 0.14 kN over 50 cycles without signs of permanent deformation (hysteresis ˂2%) (Fig. 3F and G).

### 3.3 Mechanical characterization of 3D printed magnesium-based scaffolds at rapid scaffold in vitro degradation

To assess the mechanical stability of the printed implants after degradation, uniaxial compression tests and morphology analysis were performed over 15 days on MgPSr-PCL30 immersed in PBS and lipase doped PBS (Fig. S3). MgPSr-PCL30 implants showed approximately 25% weight loss when immersed in enzymatic media for 15 days and no significant degradation when immersed in PBS (Fig. S3 A-C). Although a significant decrease in implants stiffness was observed after immersion in lipase doped PBS (Fig. S3 D), implants largely maintained their mechanical compliance (Fig. S3 E-F).

### 3.4 Ion release study

To investigate the effect of polymer masking on ceramic phase exposure, SEM analysis and release of Mg^2+^, ${\text{PO}}_{4}^{3-}$ and Sr^2+^ ions from MgPSr-PCL30 and MgP-PCL30 scaffolds were investigated in Mili-Q water and Tris-HCl solutions. Both scaffold compositions presented a highly exposed ceramic surface area (Figs. S3A–B) and a sustained ion release over 21 days (Figs. S3C–D). Mg^2+^ and ${\text{PO}}_{4}^{3-}$ ions released from the MgPSr-PCL30 scaffolds were, respectively, 0.8 and 0.42 times less than those released from the MgP-PCL30 scaffolds after 21 days immersion in Mili-Q water (Fig. S4 C). As expected, the release of Mg^2+^ ions was significantly higher in Tris-HCl, a more physiologically relevant fluid, than in Mili-Q (Fig. S4 C-D). In addition, the release of Mg^2+^ ions from the MgPSr-PCL30 scaffolds was approximately 7 times less than from MgP-PCL30, after 21 days immersion in Tris-HCl. Interestingly, no significant difference was observed between the amount of Sr^2+^ and ${\text{PO}}_{4}^{3-}$ ions released from MgPSr-PCL30 scaffolds in both Mili-Q water and Tris-HCl (Fig. S4 C-D).

### 3.5 Cytocompatibility and osteogenic potential of modified MgP constructs

eMSCs proliferated and grew faster in MgPSr-PCL30 scaffold structures, compared to those based on MgP-PCL30 or PCL alone, as revealed by live-dead staining (Fig. 3A). Quantitative live-dead staining showed that the ratio of live to dead cells after 14 days of *in vitro* culture was 99% for MgPSr-PCL30, which was 1.04 and 1.4 times higher than MgP-PCL30 and PCL alone, respectively (Fig. 3B). Additional SEM and metabolic activity analysis confirmed that the use of a solvent-based extrusion printing approach used did not affect cell activity (Fig. S5 C-D) and cell spreading and proliferation (Fig. S5A) at the PCL, MgP-PCL30, MgPSr-PCL30 implants. Furthermore, the effect of Sr^2+^ substitution and polymer modification on MgP osteogenic potential was assessed. ALP activity of the eMSCs cells on PCL, MgP-PCL30 and MgPSr-PCL30 scaffolds was evaluated in osteogenic media (Fig. 3G). After 7 days of culture, the ALP activity for the MgPSr-PCL30 scaffolds was 1.2 and 1.8 times higher than MgP-PCL30 and PCL scaffolds. Also, to assess the osteogenic potential of MgPSr-PCL30 scaffolds, after 7 days of *in vitro* culture the alkaline phosphatase activity (ALP) increased up to 2.4 U mg ^–1^ DNA for MgPSr-PCL30 in basal media which was substantially higher than for MgP-PCL30 (1.2 U mg^–1^ DNA) and PCL scaffolds alone (0.8 U mg^–1^ DNA) (Fig. 3C). Furthermore, the alp activity of MgPSr-PCL30 in basal media was 3.7 times more than in osteogenic media.

In the osteogenic media, the amount of calcium deposited on the MgPSr-PCL30 scaffolds after 30 days of culturing in osteogenic media was 1.1 and 3.2 times higher than on MgP-PCL30 and PCL scaffolds (Fig. 3H). Alizarin red staining confirmed the osteogenic properties of the MgPSr-PCL30 scaffolds (cultured in basal medium) as the average calcium deposition on MgPSr-PCL30 (277.6 μM) was 1.4 and 11.5 times higher than on MgP-PCL30 and PCL scaffolds, respectively (Fig. 3D). The calcium deposition of the MgPSr-PCL30 in basal media was 1.4 times higher than osteogenic media. Immunohistochemistry signal for osteo-nectin and type I collagen was detected in the cells attached to the printed scaffolds (Fig. S6). From the IHS signals, it was clear that the signals of osteonectin proteins increased after 21 days of *in vitro* culturing. Moreover, type I collagen was also expressed during the *in vitro* experiment, predominantly in the MgPSr-PCL30 implants, which further confirmed osteogenic properties of the MgPSr-PCL30 scaffolds.

To investigate the chemical composition of newly formed tissue after 30 days culturing MgPSr-PCL30 scaffolds, mineralization of the samples was analyzed using XRD and FTIR in both culture media (Fig. 3E, F, I, and J). XRD analysis showed two hydroxyapatite-related peaks in basal medium for MgPSr-PCL30 which could not be found in the diffraction pattern of MgP-PCL30 or PCL alone (compare to the precultured XRD results) (Fig. 3E, D). The FTIR spectra were in line with this observation, as they confirmed the presence of phosphate groups on the mineralized scaffolds of MgPSr-PCL30 in basal medium. Phosphate groups were also identified in the scaffolds doped with Sr^2+^ ions (Fig. F, G).

### 3.6 In vivo behavior of the printed MgPSr composite scaffolds

**Fig. 4:**
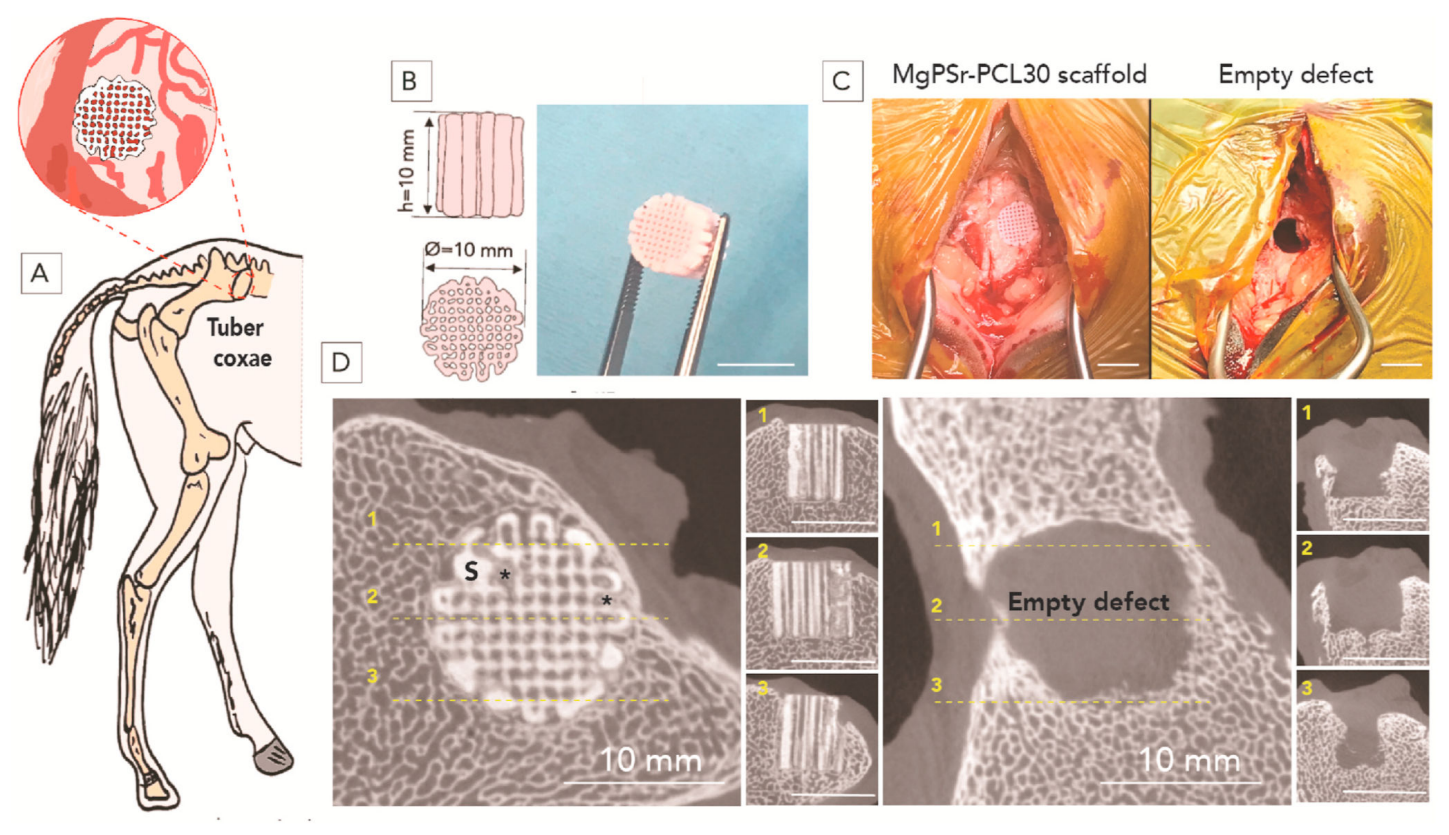
*In vivo* study preparation and implantation. A) Schematic representation of the implantation of cylindrical constructs in the equine tuber coxae. B) Drawing and photograph of the large-size printed implants (10 mm × 10 mm). C) Intraoperative views of the surgical procedure, showing empty defect and defect filled with the construct. D) μ-CT analysis of new bone formation after 6 months. Representative reconstructed images of longitudinal and transverse cross-sections of defects implanted with the MgPSr-PCL30 and empty defect after 6 months in vivo (scale bar = 10 mm).

MgPSr-PCL30 scaffolds were successfully implanted in the tuber coxae of ponies (Fig. 4A, B, and C). Micro-CT analysis revealed that the defects filled with MgPSr-PCL30 scaffolds contained 12 ± 2% newly formed bone tissue, against only 2 ± 1% in empty defects. Bone growth was observed not only at the periphery of the scaffolds but also in its center (Fig. 4D). Measured volumes of the MgPSr-PCL30 scaffolds within the created bone defects showed that 15 ± 2.7% of the scaffolds were degraded after 6 months of implantation.

**Fig. 5:**
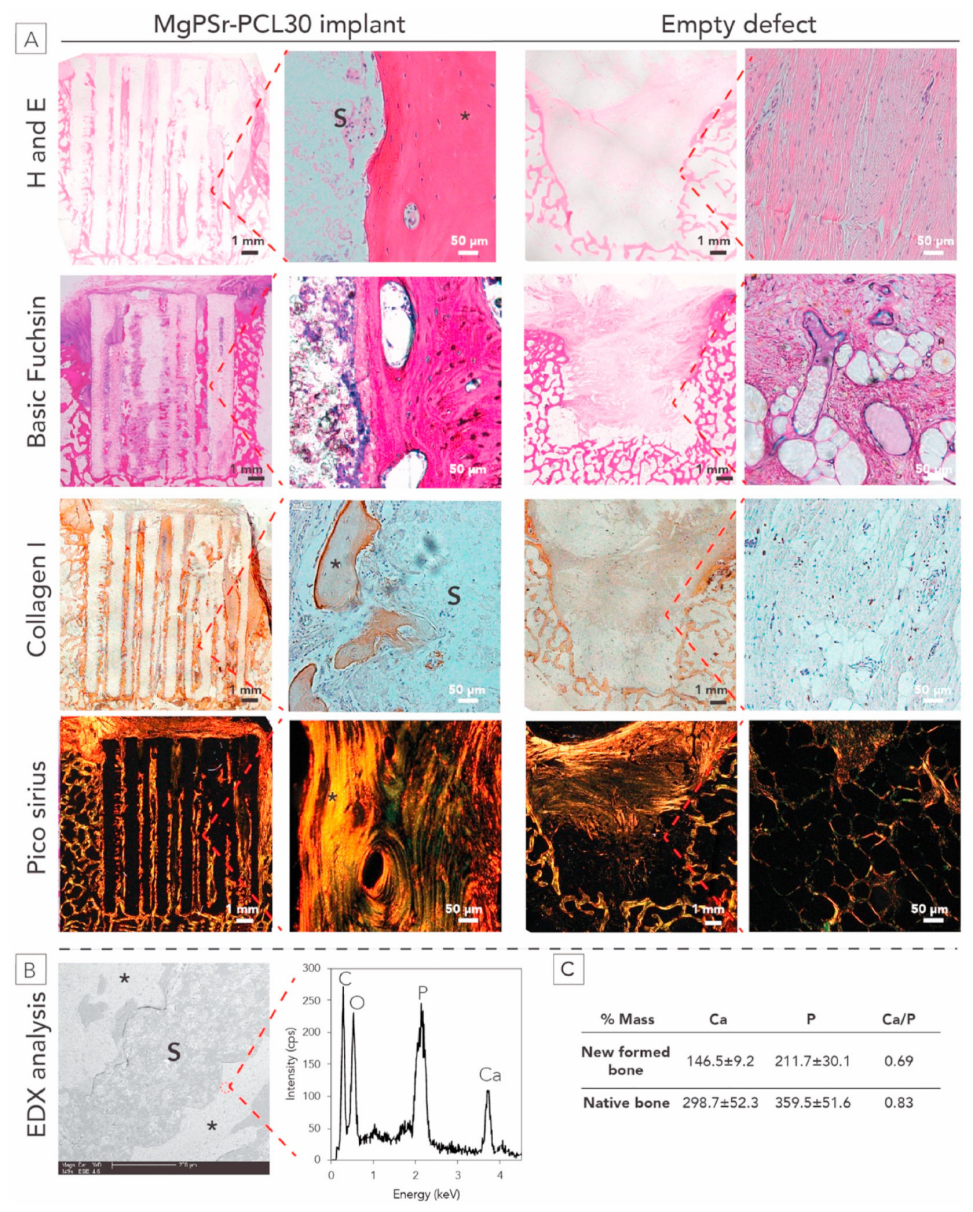
Histology assessment after 6-months *in vivo* study for the equine model. A) Histological assessment of new bone (*) within the MgPSr-PCL30 scaffolds after 6 months. Representative hematoxylin and eosin, Basic fuchsin/methylene blue-stained MMA samples, immunohistochemical staining for collagen type I (brown region), and picrosirius red–stained tissue sections of defects filled by MgPSr-PCL30 scaffolds (S) and of empty defects. The scale bar is 50 μm. B) EDX analysis of newly formed bone. Representative BSE image of newly formed bone adjacent to the scaffold strut. C) Calcium and Phosphorous analysis for newly formed bone and native one. (For interpretation of the references to color in this figure legend, the reader is referred to the Web version of this article.)

Histological analysis by H&E staining confirmed that bone defects filled with MgPSr-PCL30 composites were able to promote new bone formation (Fig. 5A), while in empty defects, no new bone growth was observed. Basic fuchsin/methylene blue-stained staining confirmed no signs of local tissue reaction or infections after 6 months of implantation (Fig. 5A). Areas with positive staining for collagen type I were located homogeneously throughout the scaffolds (Fig. 5A). Polarized light microscopy evaluation of picrosirius red-stained slides showed no significant difference between the collagen orientation in the newly formed bone and the native bone next to the scaffolds (data not shown) Mineralization of the new bone tissue was confirmed by EDX analysis (Fig. 5B). Calcium and phosphorous appeared to be homogeneously distributed (Fig. 5B) in the newly formed bone as is the case in native bone (Fig. 5C). Moreover, the calcium to phosphorous ratio is 0.69 and 0.83 for newly formed bone and native bone, respectively.

## 4 Discussion

Currently, there is an urgent need to develop patient-tailored implants to repair large bone defects. To this end, solutions must be found that combines optimal bone repair while maintaining mechanical integrity. Herein, we have developed a novel tough and bioactive material, composed of MgPSr and PCL, that can be extruded 3D printed at room temperature. The resultant 3D printed composite implants significantly enhanced the osteogenic response of mesenchymal stem cells without any osteo-inducing factors in the *in vitro* culture media and were capable of effectively repairing critical sized bone defects, while implanted in equine tuber coxae models for 6 months.

Notably, 3D-printed scaffolds with 30% PCL content (high viscosity) were readily printable at room temperature. However, when the PCL content increased to 50% (low viscosity) the 3D printing process became substantially compromised and it was difficult to achieve the needed architectural resolution for bone ingrowth which is in the range of 300–900 μm [36]. Specifically, with higher polymer content, the deposition of extruded filaments was feasible; however due to rapid solvent evaporation, they dried out, thereby losing their shape integrity and bending inwards due to their high elasticity. This strongly limits the possibility to print large and complex shaped scaffolds for further down-stream clinical applications. For this reason, we decided to only use 30% PCL to manufacture the 3D printed scaffolds.

Due to the successful incorporation of a thermoplastic PCL phase into the ceramic MgPSr phase, the resulting composite structures exhibited superior mechanical properties than pure printable ceramic materials (Table S1). The addition of 30 wt% PCL prevented the occurrence of nucleation of cracks and premature failures during loading, which is an important advantage when using ceramic-based materials at load-bearing sites. Importantly, the compressive mechanical properties of the MgPSr-PCL30 composites were in the range of native cancellous bone (Table S1) [37,38]. Moreover, since the solvent is still present during printing, the MgPSr-PCL30 struts could be fused at the junctions, which provides strong bonding between the struts. Similar findings have previously been reported for, *e.g*., PCL-HA printed scaffolds that can slightly merge at the junction of printed filaments resulting in strong bonds between the struts [39]. To evaluate the elastic properties, accumulation of the defects, and possible permanent deformation of the proposed scaffolds, mechanical properties were tested for over 50 cycles up to 0.2% strain. From the hysteresis loops, it was evident that the accumulated deformation - the shift of the hysteresis loop after 50 cycles - is less than 5% and no failure was observed. These results indicate that the 3D printed scaffolds are highly durable and thus usable as tough implants. These results are promisingsince the physiological strains imposed on human bones during daily activities are less than 0.1% with frequencies ranging from 0.5 to 2 Hz [40]. In addition, the tensile yield stress and toughness of MgPSr-PCL30 confirmed the compliance of MgPSr-PCL30 implants and their easy shapeability. Unlike the pure MgP ceramic scaffolds (Table S1) [14,22], the strength, elastic modulus, and toughness were significantly higher for the composite MgP-based implants under both compression and tensile loading. Importantly, even after accelerated *in vitro* degradation, the MgPSr-PCL30 implants were able to maintain their unprecedented compliance and load-bearing capacity.

The printability and superior mechanical properties of the MgPSr-PCL30 scaffolds is an important asset, but an often-observed challenge is the blending of osteogenic ceramics with polymers since some studies have shown that this can result in decreased osteogenic properties of the end-material as the polymers may mask the ceramic phase and compromise the release of osteogenic ions [41,42]. The ion release studies confirmed that the polymeric phase did not hamper the release of osteogenic ions from the MgPSr ceramic phase. In particular, for MgP-PCL30 scaffold, the quantity of the Mg^2+^ ions used during the synthesis was 1.3 times more than MgPSr-PCL30, resulting in a similar difference in release value. Interestingly, the presence of Sr^2+^ in MgP ceramics appears to play a role in stabilizing the MgP structures hindering the release of Mg^2+^ and phosphorous ions in both media. This could be due to Sr^+^ substituting Mg^2+^ in MgP and thereby inhibiting the release of neighboring [43–45]. Nevertheless, the eventual overall performance of the scaffolds *in vitro* and in vivo and the process of mineralization and repair of bone tissue is greatly influenced by the combined influence of bone minerals, such as Mg^2+^, Sr^2+^, and ${\text{PO}}_{4}^{2-}$.

This was also clear from our *in vitro* cell assays as hydroxyapatite precipitation was detected after 21 days of culturing on the MgPSr-PCL30 scaffold, but there is no HA formation for MgP-PCL30. We speculate that the bioactive scaffolds resulted in the release of Mg^+^ and Sr^2+^ ions due to dissolution of MgP ceramics – something which has been proven earlier to significantly affect cellular response and matrix and mineral deposition [46,47]. For instance, Mg^2+^ is one of the intra-cellular divalent cations driving cells into the S-phase and thereby enabling them to proliferate [21]. However, the clinical use of MgP ceramics is still limited due to the burst release of Mg^2+^. Such a burst release is linked to an increase in pH value above 7.4, which can comprise the bone formation process [21]. This can be counteracted by Sr^2+^ release, known to decrease pH and enhance the calcium deposition process [48].

We deliberately tested MgPSr-PCL30 scaffolds in a critical size equine model; as rodent and rabbit models are deemed not suited for this application and large animal models are seen as essential to test the feasibility of tissue engineering strategies for regenerating larger bone volumes [49]. It is well accepted that intrinsic osteoinduction properties occurring in large animal models are closer to the human body compared to small animal models [50]. Furthermore, since intrinsic osteoinduction is a long-term process, the in *vivo study* lasted 6 months to allow enough time for substantial bone formation. The scaffolds showed the ability to induce bridging of the critical-sized defects *in vivo,* and μ-CT analysis indicated that the volume of mineralized tissue in the implanted group was significantly higher than in the empty defects. Additionally, the scaffolds appeared to possess both osteoconductive and osteoinductive properties, being not only able to support bone growth surrounding the implant, but also to bridge the bone defect. There was also a significant invasion of new bone through the scaffold pores from the edges toward the center of the defect. Interestingly, EDX analysis of the newly formed bone revealed a mineral composition and Ca to P ratio similar to the native equine bone, which confirmed the osteopromotive properties of the develop scaffold materials.

## 5 Conclusion

In conclusion, we have successfully fabricated mechanical robust and osteoregenerative bone implants composed of strontium doped MgP-ceramics combined with medical-grade polycaprolactone. We identified a MgPSr/PCL composition that resulted in tough scaffolds and facilitated the osteogenic differentiation of eMSCs. Importantly, the scaffolds demonstrated improved mechanical and biological properties in comparison to pristine PCL. The MgPSr-PCL30 scaffolds, releasing of Mg^2+^ and Sr^2+^ ions, enabled eMSCs to deposit a mature bone-like matrix consisting of differentiated cells and crystalline apatite without being exposed to any additional differentiation conditions. Notably, the printed composite implants facilitated surgical handling and induced the formation of the new bone when implanted in equine tuber coxae model for 6 months, without eliciting any negative inflammatory reaction. Overall, our results showed that the addition of PCL and MgP doped with Sr^2+^ ceramics to 3D printed scaffolds could provide tough scaffolds and viable mechanism to induce bioactivity for bone tissue engineering, respectively.

## Supplementary Material

Supplementary data to this article can be found online at https://doi.org/10.1016/j.biomaterials.2020.120302.

Supplementary files

## Figures and Tables

**Table 1:** Chemical composition ratio of the reactant to synthesize MgP based powders.

	Reactant composition ratio in mole		
	MgHPO_4_·3H2O	Mg(OH)_2_	SrCO_3_
Mg_3_(PO_4_)_2_	0.6	0.3	0
Mg_2.33_Sr_0.67_(PO_4_)_2_	0.6	0.1	0.2

