# Supplementary material for: Tough magnesium phosphate-based 3D-printed implants induce bone regeneration in an equine defect model
Source: Biomaterials. Author manuscript; Available in PMC 2020 Dec 1. (PMC7116184; doi:10.1016/j.biomaterials.2020.120302)
Supplement: Supplementary files [file EMS96605-supplement-Supplementary_files.zip › 1-s2.0-S0142961220305482-mmc2.doc]

**Supplementary material**

Table S1. Comparison of the structural and mechanical properties of magnesium phosphate-based scaffolds.

| **Composition (fabrication method)** | **Structural properties** | | **Mechanical properties** | | | | **Ref** |
| --- | --- | --- | --- | --- | --- | --- | --- |
|  | **Pore size [µm]** | **Porosity [%]** | **Compressive modulus [MPa]** | **Compressive**  **Strength [MPa]** | **Tensile modulus [MPa]** | **Tensile**  **Strength [MPa]** |  |
| Magnesium phosphate (3D-printing) | 300 | - | 43 | 19 | - | - | (1) |
| Strontium modified magnesium phosphate (3D powder printing) | 21 | 15 | 10 | 30 | 10 | - | (2) |
| Magnesium phosphate (3D printing and leaching) | - | 30 | 23.9 | 3 | - | - | (3) |
| Magnesium-calcium phosphate (leaching) | - | 52-78 | - | 8.6-3.1 | - | - | (4) |
| Magnesium hydrogen phosphate (cement) | - | - | - | 10 | - | - | (5) |
| Magnesium phosphate - Calcium sulfate (cement) | - | - | - | 12 | - | - | (6) |
| Magnesium phosphate (Microwave assisted) | - | - | 200 | 12 | - | - | (7) |
| **Current work** | **490** | **40** | **36.8** | **4.3** | **24.8** | **1.5** | **-** |
| Trabecular bone | 300-600 | 75-85 | 120-1100 | 0.1-16 | - | 10-20 | (8–10) |

**Figures**

**Figure S1**. A, B) SEM image and C) X-ray diffraction pattern of MgPSr and MgP ceramics particles after annealing at 1050°C.

**Figure S2.** Photograph series of MgPSr-PCL30 scaffold under bending and torsion load.

**Figure S3.** Effect of in vitro enzymatic degradation on MgPSr-PCL30 morphological and compressive mechanical properties. A**)** Schematic figure of MgPSr-PCL30 hydrolysis in PBS and in lipase doped PBS. B) Weight loss of MgPSr-PCL30 implants in PBS and in lipase doped PBS over 15 days. C) Representative images of MgPSr-PCL30 implants after 15 days incubation in lipase doped PBS incubation. Quantification of D) elastic modulus, E) yield stress, and F) toughness of the implants as printed and after 15 days incubation in PBS and in lipase doped PBS incubation.

**Figure S4.** A and B) representative SEM images of MgPSr-PCL30 and MgP-PCL30, respectively. Ion release from 3D printed MgPSr-PCL30 and MgP-PCL30 scaffolds after immersion in C) Mili-Q water and D) Tris-HCl, respectively. Ion concentrations of Mg^2+^, PO_4_^3-^and Sr^2+^ were measured in supernatants by means of ICP-MS.

“**Figure S5.** **Cytocompatibility of 3D printed materials.** A) Representative SEM images of cell attachment to 3DP implants when cultured in basal media. Metabolic activity normalized to DNA at day 1, 7 and 14 of 3D printed constructs cultured in B) basal medium and C) osteogenic.“

**Figure S6.** Histology assessment of *in vitro* cultured 3D printed implants. Osteonectin, collagen type I positive staining after 14 and 21 days of in vitro culture in basal and osteogenic medium demonstrate osteopromotive properties of MgSr-PCL30 (S represent printed scaffold).

**References**

1.	Lee J, Farag MM, Park EK, Lim J, Yun HS. A simultaneous process of 3D magnesium phosphate scaffold fabrication and bioactive substance loading for hard tissue regeneration. Mater Sci Eng C [Internet]. 2014;36(1):252–60. Available from: http://dx.doi.org/10.1016/j.msec.2013.12.007

2.	Meininger S, Mandal S, Kumar A, Groll J, Basu B, Gbureck U. Strength reliability and in vitro degradation of three-dimensional powder printed strontium-substituted magnesium phosphate scaffolds. Acta Biomater [Internet]. 2015/12/02. 2016;31:401–11. Available from: https://www.ncbi.nlm.nih.gov/pubmed/26621692

3.	Kim JA, Lim J, Naren R, Yun HS, Park EK. Effect of the biodegradation rate controlled by pore structures in magnesium phosphate ceramic scaffolds on bone tissue regeneration in vivo. Acta Biomater [Internet]. 2016/08/25. 2016;44:155–67. Available from: https://www.ncbi.nlm.nih.gov/pubmed/27554019

4.	Wei J, Jia J, Wu F, Wei S, Zhou H, Zhang H, et al. Hierarchically microporous/macroporous scaffold of magnesium-calcium phosphate for bone tissue regeneration. Biomaterials [Internet]. 2010;31(6):1260–9. Available from: http://dx.doi.org/10.1016/j.biomaterials.2009.11.005

5.	Klammert U, Ignatius A, Wolfram U, Reuther T, Gbureck U. In vivo degradation of low temperature calcium and magnesium phosphate ceramics in a heterotopic model. Acta Biomater [Internet]. 2011;7(9):3469–75. Available from: http://dx.doi.org/10.1016/j.actbio.2011.05.022

6.	Yang G, Liu J, Li F, Pan Z, Ni X, Shen Y, et al. Bioactive calcium sulfate/magnesium phosphate cement for bone substitute applications. Mater Sci Eng C [Internet]. 2014;35(1):70–6. Available from: http://dx.doi.org/10.1016/j.msec.2013.10.016

7.	Zhou H, Agarwal AK, Goel VK, Bhaduri SB. Microwave assisted preparation of magnesium phosphate cement (MPC) for orthopedic applications: A novel solution to the exothermicity problem. Mater Sci Eng C [Internet]. 2013;33(7):4288–94. Available from: http://dx.doi.org/10.1016/j.msec.2013.06.034

8.	Lee S, Porter M, Wasko S, Lau G, Chen PY, Novitskaya EE, et al. Potential bone replacement materials prepared by two methods. Mater Res Soc Symp Proc. 2012;1418:177–88.

9.	Fiume E, Barberi J, Verné E, Baino F. Bioactive glasses: From parent 45S5 Composition to Scaffold-Assisted Tissue-Healing Therapies. J Funct Biomater. 2018;9(1).

10.	Mohamed S, Shamaz BH. Bone Tissue Engineering and Bony Scaffolds. Int J Dent Oral Heal. 2015;1(1):01–6.
